# Supplementary material for: Evolvability in the Cephalothoracic Structural Complexity of Aegla araucaniensis (Crustacea: Decapoda) Determined by a Developmental System with Low Covariational Constraint
Source: Biology (Basel). 2022 Jun 24;11(7):958. doi: 10.3390/biology11070958 (PMC9311601; doi:10.3390/biology11070958)
Supplement: Supplementary file 1 [file biology-11-00958-s001.zip › file S1.pdf]

Functional remodeling of the cephalothoracic modularity in adults of *Aegla araucaniensis* due to developmental and sexual effects

Modelling cephalothorax modularity expressions with EMMLi

The fit of different groups of developmental modularity, functional modularity given by sexual dimorphism models (bimodularity models), and anatomical multimodularity models were evaluated for the three comparison groups (all individuals, females and males). To obtain datasets of Procrustes coordinates aligned by the centroid the landmarks configurations were submitted to a Generalized Procrustes Analysis (GPA) with the tpsRelw 1.69 software (Rohlf 2003). Using these data, we compute correlations matrices of the Procrustes coordinates of 66x66 (33 x and 33 y landmark points) dimensions. Because the elements of this matrix (values of Pearson product-moment coefficient) have different levels of relationship to each other, the absolute values of these coefficients were subjected to a r-z Fisher transformation to obtain multivariate normal distributions and to control the violations of independence of related data (DeLeeuw 1983).

We compared 19 possible partition models of cephalothorax modularity. The first is a default model of non-modularity that considers the cephalothorax as a single integrated morphological unit (Fig. S1A). Three groups of bimodular models according to: (2) the developmental modularity considering the layout of cephalic and thoracic components delimited by the cervical groove (Fig. S1B), (3) functional modularity given by female sexual dimorphism (Fig. S1C) or gonadic modularity by the internal space that the reproductive system used during the gonadic cycle (*sensu* Sokolowicz et al. 2007), (4) functional modularity given by sexual dimorphism in males or agonistic modularity composed of spinous lateral and frontal processes (Fig. S1D). Each of these four models was subdivided into two submodels: a simple one with same

magnitude of correlation within both modules, and a more complex with different levels of intramodule correlation. Three anatomical multimodularity models were also tested. These models considered all possible subdivisions of six modules given by the groove pattern displayed by the dorsal surface of the thoracic module (Figs. S1E to S1G). Each multimodularity model was subdivided into four submodels that combined equal or different levels of correlation within and between modules. The number of parameters of each model (K) is given by the number of different magnitudes of correlation within and between modules (ρ values *sensu* Goswami & Finnarelli 2016), plus an additional parameter corresponding to the variance around a hypothetical correlation value of the sample (Fig. S1). The intra- and inter-module correlation values ρ approximate a normal distribution whose mean (μ_ρ) and variance (σ_ρ^2) are described in Goswami & Finarelli (2016). The parameterization of these distributions has a Log-likelihood support value given the Fisher-transformed elements of the correlation matrices (Edwards 1992). Using the obtained Log-likelihood values, we estimate the fit of each model with the Akaike Information Criterion corrected by the number of parameters for a finite sample (AICc: Hurvich & Tsai 1989). Subsequently, comparisons between model fit were made by estimating the ΔAICc or the difference between the model with the lowest AICc and the remaining models. The value of this comparison parameter allows calculating the Log-likelihood of the model, which is adjusted by the penalty due to the parameterization (Burnham & Anderson 2002). Finally, dividing each model's likelihood (i.e., $e^{\text{Model LogL}}$) by the sum of the raw data likelihoods over all model examined, it is possible to calculate the posterior probabilities of each model tested (Burnham & Anderson 2004). All analyses were performed with the EMMLi package implemented in R 3.6.0 (Goswami & Finarelli 2016).

RESULTS

The best fitting model selected by AICc for all individuals was the gonadic modularity with different correlations within each module, concentrating approximately 57% of the posterior probability of all models compared (Table S1). The ΔAICc value indicated that this model differed slightly from the equal intra-module correlation model. This was because the magnitude of within-module 1 correlation (ρ_1: 0.25) was slightly less than the correlation within module 2 (ρ_2: 0.26), and both were obviously greater than the correlation between modules (ρ_{12}: 0.19). The gonad modularity model in the females with the same intramodular correlation presented the best fit value of AICc, concentrating practically 100% of the posterior probability of the models (MPP=1.0; Table 1). The estimated intra-module correlation in this case was 0.3 for both modules ($\rho_1=\rho_2$) and 0.23 for the correlation between modules (ρ_{12}). In males, the agonistic modularity with the same intramodular correlation model had the best AICc support and a posterior probability of ~60% (Table S1). Under this model, intra- and inter-module correlation values were $\rho_1=\rho_2$: 0.23 and ρ_{12}=0.19.

REFERENCES

Burnham, K. P., and D. R. Anderson. 2002. Model selection and multimodel inference: a practical information-theoretic approach. Second Edition. Springer, New York.

Burnham, K. P., and D. R. Anderson. 2004. Multimodel inference: understanding AIC and BIC in model selection. Sociol. Methods Res. 33: 261-304.

De Leeuw, J. 1983. Models and methods for the analysis of correlation coefficients. J. Econom. 22(1-2): 113-137.

Edwards, A. W. F. 1992. Likelihood: expanded edition. The Johns Hopkins University Press, Baltimore.

Goswami, A., and J. A. Finarelli. 2016. EMMLi: A maximum likelihood approach to the analysis of modularity. Evolution. 70:1622-1637.

Hurvich, C. M., and C. L. Tsai. 1989. Regression and time series model selection in small samples. Biometrika. 76: 297-307.

Rohlf, F. J. 2003. tpsRelw, relative warps analysis, version 1.69. Departament of Ecology and Evolution, State University of New York at Stony Brooks.

Sokolowicz, C. C., L. S. López-Greco, R. Gonçalves, and G. Bond-Buckup. 2007. The gonads of *Aegla platensis* Schmitt (Decapoda: Anomura: Aeglidae): a macroscopic and histological perspective. Acta Zoologica. 88:71-79.

Table and Figure Legends

Figure S1. Models of bi- and multiple cephalothorax modularity tested in this study. A brief structural and parametric characterization of each type of model is included.

Table S1. Results of the different modularity models compared under a maximum likelihood approach. Bold indicates the best fit model in each comparison group (MPP: Model Posterior Probability).
