# Supplementary figures and images for: Evolvability in the Cephalothoracic Structural Complexity of Aegla araucaniensis (Crustacea: Decapoda) Determined by a Developmental System with Low Covariational Constraint
Source: Biology (Basel). 2022 Jun 24;11(7):958. doi: 10.3390/biology11070958 (PMC9311601; doi:10.3390/biology11070958)

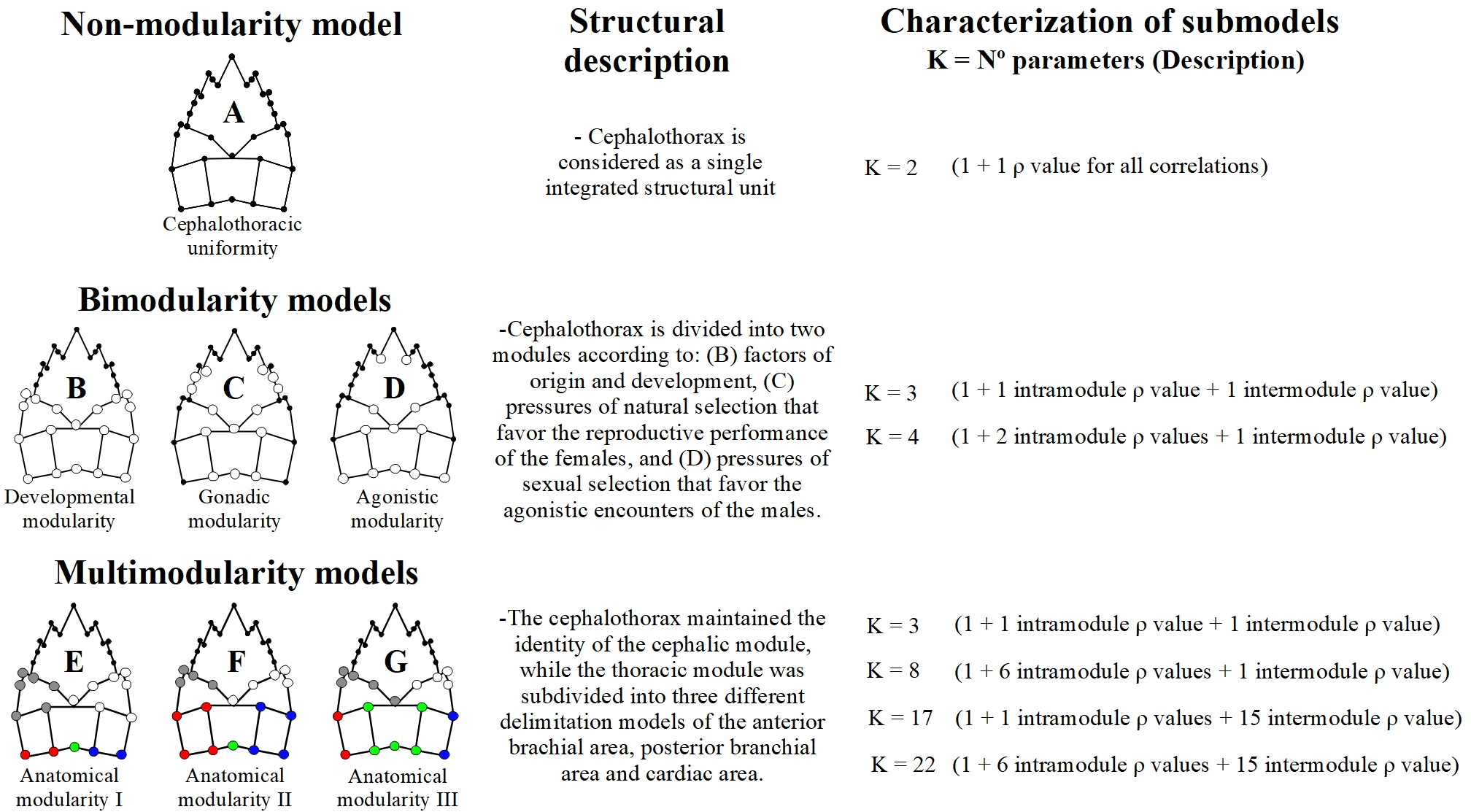

Supplement: Supplementary file 1 [file biology-11-00958-s001.zip › Figure S1.jpg]
